# Mechanism Study on Nanoparticle Negative Surface Charge Modification by Ascorbyl Palmitate and Its Improvement of Tumor Targeting Ability

**DOI:** 10.3390/molecules27144408

**Published:** 2022-07-09

**Authors:** Lin Li, Hongliang Wang, Jun Ye, Yankun Chen, Renyun Wang, Dujia Jin, Yuling Liu

**Affiliations:** 1 State Key Laboratory of Bioactive Substance and Function of Natural Medicines, Institute of Materia Medica, Chinese Academy of Medical Sciences & Peking Union Medical College, Beijing 100050, China; llin@imm.ac.cn (L.L.); wanghl@imm.ac.cn (H.W.); yelinghao@imm.ac.cn (J.Y.); wry@imm.ac.cn (R.W.); djjin@imm.ac.cn (D.J.); 2 Beijing Key Laboratory of Drug Delivery Technology and Novel Formulation, Institute of Materia Medica, Chinese Academy of Medical Sciences & Peking Union Medical College, Beijing 100050, China; 3 Beijing Wehand-Bio Pharmaceutical Company Limited, 30 Tianfu Street, Beijing 102600, China; yankun.chen@wehandbio.com

**Keywords:** ascorbyl palmitate, lipid nanoparticles, surface charge, molecular dynamics simulation, physicochemical characteristic, tumor targeting

## Abstract

Surface charge polarity and density influence the immune clearance and cellular uptake of intravenously administered lipid nanoparticles (LNPs), thus determining the efficiency of their delivery to the target. Here, we modified the surface charge with ascorbyl palmitate (AsP) used as a negatively charged lipid. AsP-PC-LNPs were prepared by dispersion and ultrasonication of AsP and phosphatidylcholine (PC) composite films at various ratios. AsP inserted into the PC film with its polar head outward. The p*K*_a_ for AsP was 4.34, and its ion form conferred the LNPs with negative surface charge. Zeta potentials were correlated with the amount and distribution of AsP on the LNPs surface. DSC, Raman and FTIR spectra, and molecular dynamics simulations disclosed that AsP distributed homogeneously in PC at 1–8% (*w*/*w*), and there were strong hydrogen bonds between the polar heads of AsP and PC (PO^2−^), which favored LNPs’ stability. But at AsP:PC > 8% (*w*/*w*), the excessive AsP changed the interaction modes between AsP and PC. The AsP–PC composite films became inhomogeneous, and their phase transition behaviors and Raman and FTIR spectra were altered. Our results clarified the mechanism of surface charge modification by AsP and provided a rational use of AsP as a charged lipid to modify LNP surface properties in targeted drug delivery systems. Furthermore, AsP–PC composites were used as phospholipid-based biological membranes to prepare paclitaxel-loaded LNPs, which had stable surface negative charge, better tumor targeting and tumor inhibitory effects.

## 1. Introduction

Phospholipid-based biological membranes (PBBM) are classical constructions similar in structure with biological membranes. Such structures are frequently seen in various drug carriers, such as liposomes [1,2,3], ethosomes [4,5,6,7], solid lipid nanoparticles [8,9,10,11], micelles [12,13,14,15], nanoemulsions [16,17], etc. Moreover, PBBM are highly biocompatible and can encapsulate various drugs, such as hydrophobic and hydrophilic molecules, and biomolecules such as DNA or RNA, which have attracted clinical and industrial attention in recent years [18,19,20,21]. Moreover, such structures have been shown to improve the bioavailability of active pharmaceutical ingredients (API) and help reduce drug-related morbidity [21]. However, complex biological barriers, such as opsonization and sequestration by the mononuclear phagocyte system (MPS) and target-specific cellular uptake obstruction, increase the distribution and elimination of drug-loading particles at non-target sites in vivo. These impediments lower the cumulative drug quantity reaching the disease targets and overall drug efficacy [22]. Thus, it is vital to optimize the surface properties to overcome biological barriers for drug carriers to have a high degree of application specificity [23].

Surface properties, such as charge and hydrophobicity, markedly influence the protein adsorption, immune clearance, and cellular uptake behavior of the intravenously delivered nanocarriers, by extension indirectly influencing drug pharmacokinetics and bio-distribution [24]. Many recent studies have demonstrated that surface charge polarity and density play important roles in nanocarrier performance [22,25,26,27]. At the same size and hydrophobicity, nanocarriers with positive surface charge more effectively adsorbed plasma proteins and had faster blood vessel clearance rates than negatively charged nanocarriers. The higher the charge density on the surface, the more plasma proteins adsorbed [25,28]. However, nanocarriers with neutral, zwitterionic, or negative surface charge adsorb less protein and have longer circulation times than those with positive surface charge [24,27,29,30,31]. On the other hand, nanocarriers with positively charged surfaces are more likely to be taken up by cells [26]. Nanocarriers with negative surface charge also have good cellular internalization, and due to prolonged blood circulation time, they are more effectively distributed in tumors than nanocarriers with positive surface charge of the same size [32]. It was found that long-circulating, non-targeted gold-bearing PEGylated liposomes with neutral surfaces were accumulated in the tumor stroma and did not internalize within the tumor cells as expected [33]. These studies suggest that nanocarriers with negative surface charge should have certain advantages in overcoming biological barriers and enhancing their delivery efficiency to targeted tumor cells. In any case, surface charge is an important factor affecting the delivery efficiency of intravenously delivered nanocarriers to their targets.

**Figure 1:**
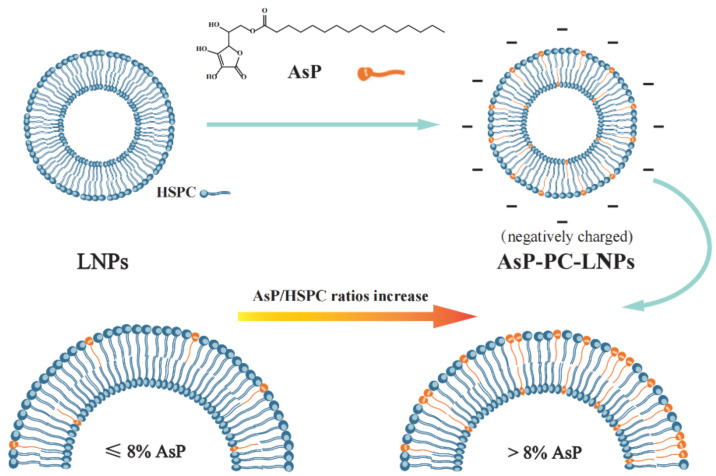
Schematic illustration of the mechanism of liposome surface charge modification by ascorbyl palmitate (AsP).

The precise regulation of nanocarrier surface charge polarity and density for specific drug delivery remains a challenge. Charged lipids are important materials for the surface charge modification. The charged lipid nanoparticles were prepared by mixing charged lipids and phospholipids at various ratios to modify and regulate the surface charge [34,35,36]. The dedicated charged lipids were either produced synthetically or semi-synthetically or derived from natural resources. However, there are few injectable charged lipids suitable for nanocarrier surface charge modification at the moment. Ascorbyl palmitate (AsP, molecular structure shown in Figure 1) is a palmitic acid ester of ascorbic acid. It is safe and effective as a lipophilic antioxidant in food processing. It was found that incorporation of AsP into the PBBM formulations can increase the negative zeta potential of nanocarriers and stabilizes them [37,38]. Moreover, AsP-modified PBBM can enhance specific binding to tumor cells, potentiate the delivery of encapsulated paclitaxel, and increase selective cytotoxicity [38]. Nevertheless, few comprehensive investigations have been conducted on the mechanism by which AsP modifies the PBBM negative surface charge. In addition, the molecular-level interactions between AsP and phospholipids have not been elucidated. Moreover, it is also unknown how the physicochemical properties of lipid nanocarriers change with the proportion of AsP.

The aims of this study were to elucidate the mechanism of negative surface charge modification of the phospholipid-based biological membranes by AsP, determine the influence of AsP on the physicochemical properties of lipid nanocarriers, clarify the interaction between AsP and phosphatidylcholine (PC), and provide a theoretical foundation for the rational use of AsP in lipid nanocarrier surface modification. To these ends, p*K*_a_ of AsP was measured to assess its ionization in water. AsP–PC lipid nanocarriers were prepared by various AsP/PC composites. Particle size distribution and zeta potential were measured. DSC, Raman, and FTIR spectra were plotted to evaluate the impact of AsP on the physicochemical properties of liposome. Molecular dynamics (MD) studies were performed to simulate 3D models of AsP–PC composites and explain molecular-level interactions between AsP and PC at various ratios. Furthermore, paclitaxel (PTX) loaded lipid-nanoparticles (PTX-LNPs) were constructed to verify the physicochemical properties and in vivo tumor-targeting ability of PBBM modified by AsP.

## 2. Materials and Methods

### 2.1. Materials

Ascorbyl palmitate (6-*O*-palmitoyl-*L*-ascorbic acid, 99.1% purity) was purchased from Sigma-Aldrich Corp. (St. Louis, MO, USA). Hydrogenated soy phosphatidylcholine (HSPC, 98.6% purity) was obtained from A.V.T. Pharmaceutical Co., Ltd. (Shanghai, China). Medium-chain triacylglycerol (MCT) was purchased from SHINSUN Pharmaceutical Co., Ltd. (Tieling, Liaoning, China). Paclitaxel (PTX, 99.0% purity) was purchased from Guilin Huiang Biochemistry Pharmaceutical Co., Ltd. (Guilin, Guangxi, China). Paclitaxel injection (5 mL:30 mg), which consists of PTX, Kolliphor EL, dehydrated citric acid and dehydrated ethanol, was purchased from Beijing Union Pharmaceutical Factory (Beijing, China). High pressure liquid chromatography (HPLC)-grade methanol was procured from Fisher Scientific (Fair Lawn, NJ, USA). Analytical-grade sodium dihydrogen phosphate and disodium hydrogen phosphate were purchased from Sinopharm Chemical Reagent Co., Ltd. (Shanghai, China). HPLC-grade 1,4-dioxane was obtained from Acros Organics (Geel, Belgium). All other reagents were analytical grade.

#### 2.1.1. Cell Culture

4T1 and MDA-MB-453 cells were purchased from the Cell Resource Center, Peking Union Medical College (Beijing, China), and cultured in Dulbecco’s Modified Eagle’s Medium with a high glucose concentration (4.5 g/L, DMEM-H, Hyclone, Logan, UT, USA) supplemented with 10% fetal bovine serum (Pan-biotech, Aidenbach, Germany) and 1% penicillin/streptomycin (Gibco, Thermo Fisher Scientific, Waltham, MA, USA) under 5% CO_2_ at 37 °C.

#### 2.1.2. Animals

Balb/c mice (females, 16–18 g) were purchased from Beijing Vital River Laboratory Animal Technology (Beijing, China) and were raised at the Institute of Material Medica, Chinese Academy of Medical Sciences and Peking Union Medical College (Beijing, China). The experiments were performed under the approval of the Laboratory Animal Care and Use Committee of the Peking Union Medical College (project identification code 3719). All animal experiments were performed in accordance with the guidelines of laboratory animals–guidelines for ethical review of animal welfare (GB_T 35892.2018), for the welfare of the animals.

### 2.2. Ascorbyl Palmitate pK_a_ Measurement

The p*K*_a_ for AsP was measured by potentiometric titration over a wide pH range in a Sirius T3 apparatus (Sirius Analytical Instruments Ltd., East Sussex, UK) fitted with a glass Ag/AgCl pH electrode, a turbidity sensor, and a precision microdispenser. The p*K*_a_ values were represented by the pH of potential mutations during acid–base titration [39,40]. For poorly water-soluble compounds, the apparent p*K*_a_ values (p_s_*K*_a_) were measured in co-solvents by mixing water and miscible solvent at various ratios. Then, the p*K*_a_ values in water were determined by extrapolation to zero co-solvent in a Yasuda–Shedlovsky fitting curve [39,40,41]. For AsP, the pH-metric p_s_*K*_a_ method was used. All titrations were conducted using co-solvents of 50–60% (*v/v*) 1,4-dioxane and 0.15 M KCl at 20–25 °C with argon gas protection. Sample solutions were pre-acidified with 0.5 M HCl. Standardized 0.5 M KOH was the titration reagent. The titration pH range was from 2.0 to 9.5.

### 2.3. AsP–PC Composite Preparation

The solvent evaporation method was used to prepare AsP/HSPC composites (AsP-PC). Briefly, AsP/HSPC (*w/w*) at 1%, 4%, 8%, 12%, and 20% were dissolved in methanol, and the solid films of AsP–PC composites were obtained by vacuum distillation to remove the solvent in a rotary evaporator (RE-3000; Shanghai Yarong Bio-Chem Instruments, Shanghai, China). Then they were desiccated under vacuum at room temperature overnight.

### 2.4. AsP–PC Lipid Nanoparticles Preparation

AsP–PC lipid nanoparticles (AsP-PC-LNPs) were prepared by film dispersion and ultrasonication. In film dispersion, AsP–PC composites with various ratios were hydrated with phosphate-buffered saline (PBS; 10 mM; pH 7.0) by stirring at 55 °C for 30 min. The suspensions were sonicated by probe in an ultrasonic homogenizer (SCIENTZ-IIE; Ningbo Scientz Biotechnology Co., Ltd., Ningbo, Zhejiang, China) at 150 W for >10 min to obtain nanoscale AsP-PC-LNPs. AsP-free lipid nanoparticles (AsP-free-LNPs) were prepared by HSPC alone with the same method.

### 2.5. Zeta Potential Measurement of AsP-PC-LNPs

AsP-PC-LNPs and AsP-free-LNPs were diluted with appropriate volumes of purified water (1/100, *v/v*) for zeta potential measurement. Their zeta potentials were evaluated with a NICOMP 380 ZLS Zeta Potential/Particle Sizer (PSS Nicomp, Santa Barbara, CA, USA), and their mean particle diameters and size distributions were measured by dynamic light scattering.

### 2.6. DSC Measurements

Appropriate amounts of water were added to AsP–PC composites (1/5, *w/w*) and sonicated at 50 W for 10 min in a bath sonicator (KQ-50B; Kunshan Ultrasonic Instruments Co., Ltd., Kunshan Jiangsu, China) at 65–75 °C to obtain a sufficiently high concentration of AsP–PC hydrates for the DSC measurement of AsP-PC-LNPs [42]. The phase transition behaviors and calorimetric data for AsP–PC hydrates with various AsP/HSPC ratios (1%, 4%, 8%, 12%, and 20% *w/w*) were compared by differential scanning calorimetry (DSC) with a DSC 6200 (Seiko Instruments, Tokyo, Japan). The samples were sealed in standard aluminum crimp cells. An empty cell was the reference. The samples were heated from 20 °C to 80 °C at a rate of 1 °C/min. Nitrogen was the shielding gas, and its flow rate was 60 mL/min.

### 2.7. Raman Spectroscopy

For the Raman spectra measurements of AsP-PC-LNPs, appropriate amounts of water were added to AsP–PC composites (1/10, *w/w*) and the aforementioned methods were used to prepare the AsP–PC hydrates. The Raman spectra of AsP–PC hydrates with various AsP/HSPC ratios (4%, 8%, and 20% *w/w*) were recorded in the Raman shift range of 50–3500 cm^−1^ under a DXR Raman microscope (Thermo Fisher Scientific, Waltham, MA, USA). The laser source had 780 nm excitation wavelength, and a 10 × confocal microscope objective was used. The spectral resolution was 1.5 cm^−1^. Measurements were made at 24 mW laser power, 50 μm slit width, and 10 s integration time. Each sample had five cumulative exposures.

### 2.8. FTIR Spectroscopy

FTIR spectra of dry AsP–PC composites with various AsP/HSPC ratios (4%, 8%, and 20% *w/w*) were recorded under the same conditions at room temperature with a Fourier transform infrared spectrometer (Nicolet 5700 FTIR spectrometer, Thermo Fisher Scientific, Waltham, MA, USA). The scan range was 4000–400 cm^−1^, and the resolution was 4.0 cm^−1^. The samples were compressed into KBr tablets, and 64 scans were needed. Appropriate amounts of water were added to AsP–PC composites (1/10, *w/w*) and the aforementioned methods were used to prepare the AsP–PC hydrates for FTIR spectra measurement of AsP-PC-LNPs under an infrared microscope (Nicolet Centaur μs, Thermo Fisher Scientific, Waltham, MA, USA) at 8.0 cm^−1^ resolution, and 100 scans were required.

### 2.9. Materials Studio Simulations of Interactions between AsP and PC

Interactions between AsP and PC were analyzed by molecular dynamics simulations in Materials Studio Software (MS, version 8.0, Accelrys Inc., San Diego, CA, USA). The AsP and HSPC molecules were built and geometrically optimized. The models of AsP–PC composites at various AsP/HSPC ratios were configured in a cubic unit cell with 1 g·cm^−3^ density by the Amorphous Cell module. Two models of AsP–PC composites at the molecular AsP/HSPC ratios of 4:27 and 10:27 n/n, respectively, were constructed by referencing the weight ratios of 8% and 20% (*w/w*). A single AsP component model with ten molecules was simulated for comparison. Before the MD simulations, the cell geometry was optimized with the conjugate gradient algorithm, and the cvff_nocross_nomorse force field was suitable. MD simulations were performed at ultrafine quality using the FORCITE module with a consistent-valence force field (CVFF) [43]. Temperature and pressure were controlled by setting a nose thermostat and Berendsen barostat, respectively. Electrostatic interactions and van der Waals terms were calculated by the Ewald and atom-based summation methods, respectively. The process of molecular dynamics simulations was performed as follows. The isothermal-isobaric ensemble (NPT) process was simulated at 308 K and 1 atm with a 1 fs time step and 50 ps total simulation time. The canonical ensemble (NVT) process was simulated at 308 K with a 1 fs time step and 100 ps total simulation time. Then, the NPT ensemble was conducted at 278 K (normal room temperature) and 1 atm and 50 ps total simulation time. After the simulations, the hydrogen bonds for intermolecular and intramolecular interactions were calculated. The maximum hydrogen acceptor distance was set to 2.5 Å.

### 2.10. Preparation of PTX–LNPs

**Figure 2:**
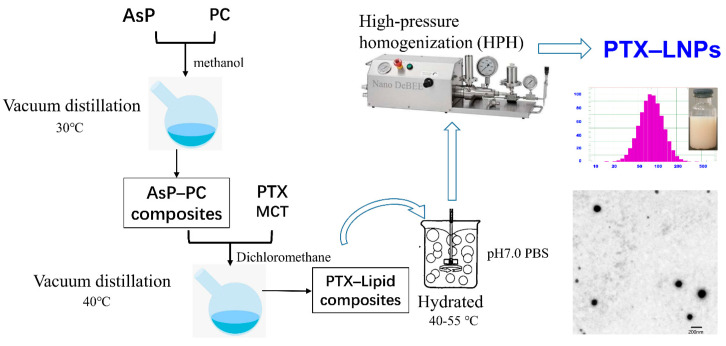
Main preparation process of PTXLNPs.

The main preparation processes of PTX–LNPs are shown in Figure 2. To prepare the PTX-LNPs, AsP 80 mg and PC 1000 mg were dissolved in methanol. Next, the organic solvent of the obtained clear lipid solution was evaporated by using a rotary evaporator at 30 °C, obtaining the AsP–PC composites. Then, medium chain triglycerides (MCT) 900 mg and PTX 20 mg were placed into the above container and dissolved in a specific volume of dichloromethane. Next, the dichloromethane was evaporated with the above method at 40 °C to obtain the PTX–lipid composites. Next, the PTX–lipid composites were hydrated with 50 mL of an aqueous solution of PBS 10 mM (pH = 7.0, with 2.5% glycerin). The hydration of the PTX–lipid composites was performed by stirring at 45 °C for 30 min. Moreover, in order to generate uniform populations, PTX–LNPs were obtained by homogenization in a high-pressure homogenizer (Nozzle Z5, Nano DeBEE, Bee International, South Easton, MA, USA). In addition, blank-LNPs without PTX were prepared by similar methods.

### 2.11. Physicochemical Characterization of PTX–LNPs

#### 2.11.1. Morphology and Particle Size and Zeta Potential

The morphological evaluation of PTX–LNPs was carried out by transmission electron microscopy (TEM, Hitachi H-7650, Tokyo, Japan) [8]. Two drops of the PTX–LNPs were deposited on a film-coated copper grid and negatively stained with 1% (*w*/*v*) phosphotungstic acid for 5 min before observation under an electron microscope [44].

The average particle size, polydispersity index (PDI), and zeta potential of the prepared PTX–LNPs were measured by photon correlation spectroscopy using a NICOMP 380 ZLS Zeta Potential/Particle Sizer (PSS Nicomp, Santa Barbara, CA, USA). The samples were diluted with water (1:200), and the measurements were performed at 25 °C. The scattering angle for measurement was 90°. To measure the particle size and PDI, the samples were placed in disposable polystyrene cells. For the zeta potential measurements, the samples were placed in disposable plain folded capillary zeta cells. All experiments were performed in triplicate, and the data were analyzed using built-in software. The results are represented as the mean ± standard deviation.

#### 2.11.2. Drug Loading (DL) and Encapsulation Efficiency (EE)

The DL and EE of PTX incorporated in PTX–LNPs were determined by HPLC using an Agilent 1200 HPLC system (Agilent Technologies, Santa Clara, CA, USA). For the analysis of PTX, a ZORBAX Eclipse XDB-C18 column (4.6 mm × 150 mm, 5 μm, Agilent Technologies, Santa Clara, CA, USA) was selected. The mobile phase was composed of methanol-water-acetone at a volume ratio of 23:41:36 and pumped at a flow rate of 1.2 mL/min. The detection wavelength was set at 227 nm, and the column temperature was set at 35 °C. The HPLC analysis method was validated and met the methodological requirements. Prior to the total PTX content analysis, 0.5 mL PTX-LNPs were dissolved in methanol-glacial acetic acid mix solvents (200:1, *v*:*v*), making sure to dissolve to 25 mL, then filtered using a 0.22 µm polyvinylidene fluoride syringe filter.

The entrapment efficiency of PTX in PTX–LNPs was determined by a mini-column centrifugation method using a 5 mL plastic syringe filled with Sephadex G-50 gel [45]. Briefly, Sephadex G-50 gel was allowed to swell in the water. Additionally, a cotton mass was placed in the cylinder of the syringe, and the mini-column was inserted into a centrifugation tube and then spun for 3 min at 2000 rpm to remove water. Then, 0.5 mL PTX–LNPs was introduced into the mini-column and then centrifuged at 2000 rpm for 3 min. This operation was repeated another 3 times. The eluted sample, which contained entrapped PTX, was dissolved in methanol-glacial acetic acid mix solvents (200:1, *v*:*v*) and then analyzed for PTX assay using HPLC method.

The DL and EE (%) of PTX–LNPs were estimated by using the following equations.

(1) $$\mathrm{Drug}\;\mathrm{Loading}\;\left(\mathrm{DL},\;\%\right)=\frac{\mathrm{amount}\;\mathrm{of}\;\mathrm{encapsulated}\;\mathrm{PTX}\;\left(\mathrm{mg}\right)}{\mathrm{total}\;\mathrm{amount}\;\mathrm{of}\;\mathrm{PTX}-\mathrm{LNPs}\;\left(\mathrm{mg}\right)}\times 100\%$$

(2) $$\mathrm{Encapsulation}\;\mathrm{Efficiency}\;\left(\mathrm{EE},\;\%\right)=\frac{\mathrm{amount}\;\mathrm{of}\;\mathrm{encapsulated}\;\mathrm{PTX}\;\left(\mathrm{mg}\right)}{\mathrm{total}\;\mathrm{amount}\;\mathrm{of}\;\mathrm{PTX}\;\left(\mathrm{mg}\right)}\times 100\%$$

### 2.12. In Vitro Cytotoxicity Analysis

The cytotoxicity of the PTX–LNPs, blank-LNPs, and PTX injection against the MDA-MB-453 cell line were measured using the CCK8 assay. Briefly, the cells were seeded into 96-well plates at a cell density of 9 × 10^4^ cells/mL (100 μL/well). After 24 h of incubation, the cells were further incubated for another 72 h in fresh DMEM-H complete medium containing 0.001, 0.005, 0.01, 0.02, 0.05, and 0.2 μg/mL PTX–LNPs, PTX injection, or the blank-LNPs, respectively. At the above-mentioned time point, the culture medium was replaced by the CCK8 solution. The absorbance values of each well were measured at 450 nm and referenced with the absorbance values at 620 nm using a microplate reader (Multiskan FC, Thermo Scientific, Waltham, MA, USA).

### 2.13. In Vivo Anti Breast Cancer Effect of PTX–LNPs

The in vivo anti breast cancer efficacy was evaluated in female BALB/c mice bearing 4T1 orthotopic breast cancer. To establish the above models, 1 × 10^6^ 4T1 cells were injected (day 0) into the number 4 mammary fatty pads of BALB/c mice. Then, 15 tumor-bearing mice were randomly and equally divided into 3 groups: control group (PBS), PTX injection group, and PTX–LNPs group. On days 2, 5, and 8, PBS, 10 mg/kg PTX injection, or 10 mg/kg PTX-LNPs was injected into the caudal vein. The body weights of the mice were recorded 3 times every week after tumor-bearing. When tumors were visible (on day 14 after cell injection), the tumor volumes were measured every day by calipers and calculated using the following formula: tumor volume = 0.5 × length × width^2^. On day 25, the mice were euthanized, and tumors were removed and weighed.

In order to observe the tumor targeted efficacy of the LNPs, DiR (1,1′-dioctadecyl-3,3,3′,3′-tetramethylindotricarbocyanine iodide) fluorescence imaging was performed with IVIS Spectrum CT (Caliper-Perkin Elmer, Waltham, MA, USA). Briefly, on the 20th day after tumor-bearing when tumor volume was above 800 mm^2^, the mice were separated into two groups and injected with DiR injection or DiR-LNPs into the caudal vein, respectively. At 2, 4, 8, 12, and 24 h post-injection, the mice were anesthetized with isoflurane and subsequently imaged with the IVIS system. A filter setting for DiR detection was fixed at 745 nm for excitation and 800 nm for emission. Average luminescence intensity (photon/s) was quantified by analyzing the tumor region with a software package.

### 2.14. Statistical Analysis

The statistical difference between the treatments was evaluated using the analysis of variance (ANOVA) test. Data are reported as mean ± standard deviation (SD). All data obtained were analyzed using GraphPad Prism software version 8.0 (GraphPad Software Inc., CA, USA).

## 3. Results and Discussion

### 3.1. pK_a_ of AsP

**Figure 3:**
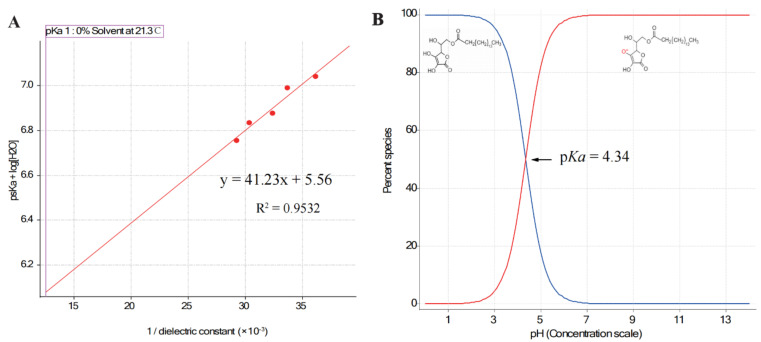
(**A**) The Yasuda–Shedlovsky curve extrapolated by fitting the p_s_*K*_a_ test values (*n* = 5); (**B**) distribution of species of AsP, an acid with p*K*_a_ = 4.34.

The p*K*_a_ value of AsP was 4.34, which was calculated by extrapolation of the Yasuda–Shedlovsky curve to 0% solvent obtained by fitting the apparent p*K*_a_ (p_s_*K*_a_) test values (Figure 3A). This conformed with the value 4.45 calculated by MavinSketch v. 6.0.0 Software (ChemAxon, Budapest, Hungary). As shown in Figure 3B, the AsP species distribution indicated that nearly all of the AsP was ionized at pH 7.0. The ionized AsP was the substance basis for modifying the negative surface charge of LNPs at neutral pH.

### 3.2. Zeta Potential Values of AsP-PC-LNPs

The zeta potential values and particle sizes of AsP-PC-LNPs with various AsP/HSPC ratios are listed in Table 1. All LNPs had similar nanoscale particle sizes. The surface charge of AsP-free-LNPs prepared by HSPC alone was almost electrically neutral. The negative zeta potentials of the AsP-PC-LNPs increased with AsP proportion at 1–8% (*w*/*w*). When the AsP/HSPC ratio was >8% (*w/w*), the negative surface potential no longer significantly changed with increasing AsP proportion. The results show that AsP confers a negative surface charge to the LNPs, and the zeta potential was correlated with the amount and distribution of AsP on the LNPs surface. As the LNPs had a limited surface area, with excessive AsP in the lipid films (>8%, *w/w*), either the AsP reached saturation on the surface or the AsP distribution form substantially changed.

### 3.3. Phase Transition Temperature Determination by DSC

DSC was conducted to determine the phase transition temperatures of AsP–PC hydrates with various AsP proportions [46]. The main phase transition reflected the change from a gel phase with ordered lipid chains to a fluid liquid–crystalline phase with disordered lipid chains [42,46,47]. Molecular interactions and head group hydration also participate in chain melting transition [46]. For mixtures of phospholipids and exogenous substances, narrow DSC peaks indicate good compositional cooperativity [26].

**Figure 4:**
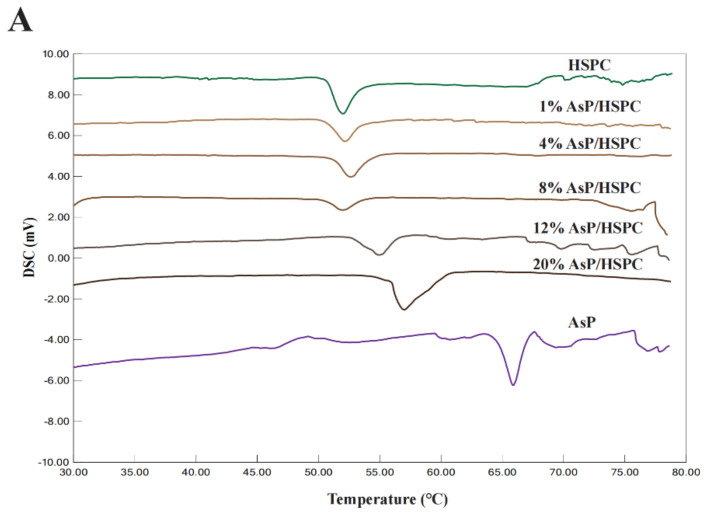

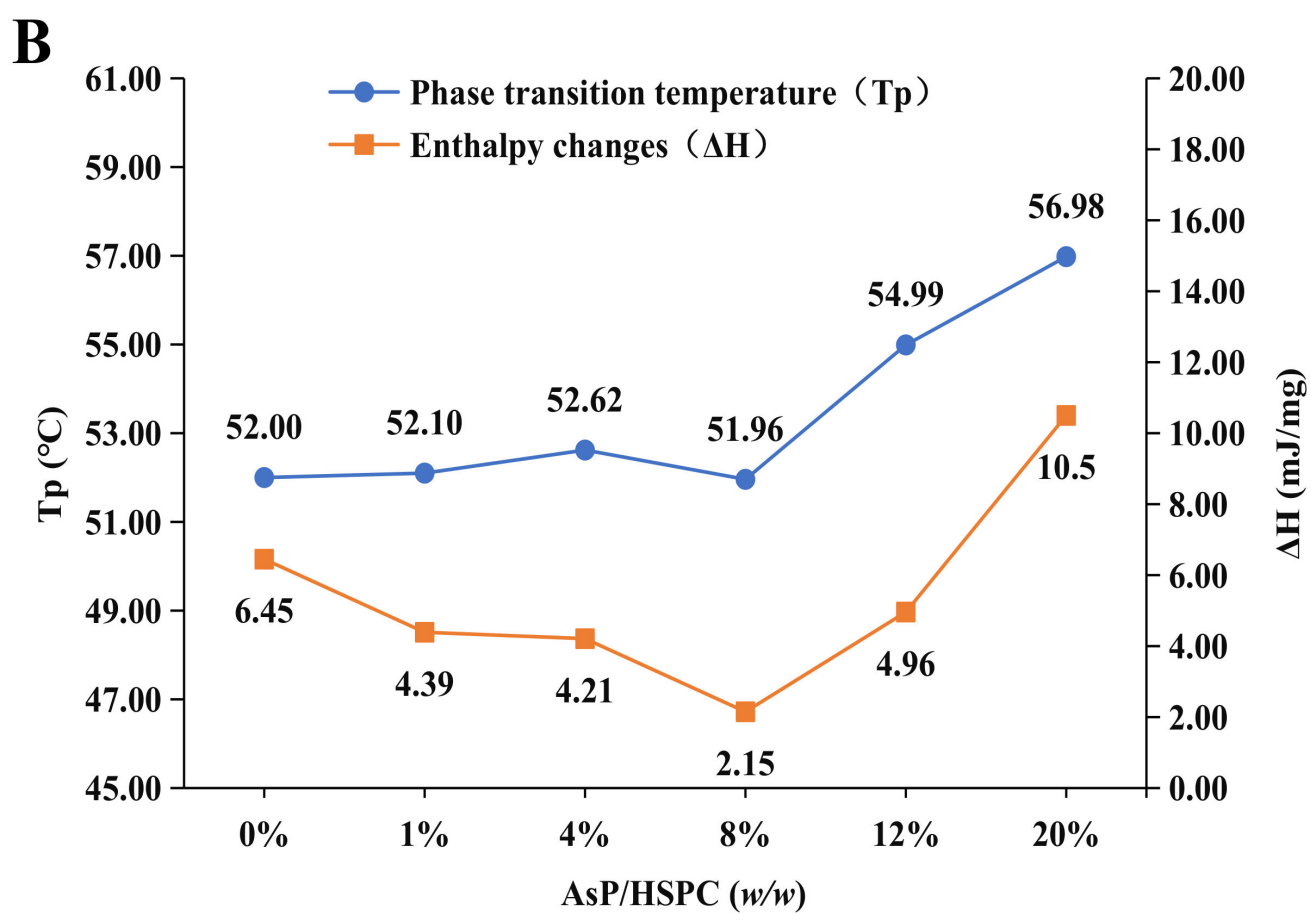
(**A**) DSC thermograms of AsP–PC hydrates with various AsP/HSPC ratios (*w/w*); (**B**) the main phase transition temperatures (Tp) and enthalpy changes (ΔH) of AsP–PC hydrates.

The DSC profiles of AsP–PC hydrates with various AsP/HSPC ratios are shown in Figure 4A. The peak temperatures of the main phase transition (Tp) and the enthalpy changes (ΔH) are depicted in Figure 4B. Pure HSPC had a main transition temperature of 52.0 °C, and no pretransition was observed (Figure 4A). AsP had a higher main transition temperature than HSPC. The main phase transition temperatures and endothermic peak widths for AsP–PC hydrates in the 1–8% (*w/w*) AsP/HSPC ratio range did not markedly differ from those for pure HSPC. Thus, AsP was uniformly dispersed in the lipid hydrates and had a good cooperativity with HSPC. Moreover, ΔH decreased with gradual increase in AsP/HSPC ratio at 1–8% (*w/w*), indicating that a small amount of uniformly dispersed AsP interferes with the ordered arrangement of the polar ends of the phosphoester film. AsP increase within this range reduces the latent heat required for the phase transition of AsP–PC hydrates.

The main transition peak width of the AsP–PC hydrates gradually increased when the AsP/HSPC ratio was >8% (*w/w*). Tp shifted to a higher temperature, and the phase change enthalpy increased (Figure 4B). Therefore, the AsP–PC cooperativity decreased and the lipid chain ordering enhanced in water when the AsP/HSPC ratio was >8% (*w/w*). This suggests that the excessive AsP strengthens the intermolecular interactions, as there are enough AsP molecules with strong polar heads in the AsP–PC hydrates. This mechanism makes the lipid chains compact and causes an increase in the latent heat of phase transition and heterogeneous AsP distribution in the AsP–PC hydrates.

### 3.4. Raman Spectroscopy

**Figure 5:**
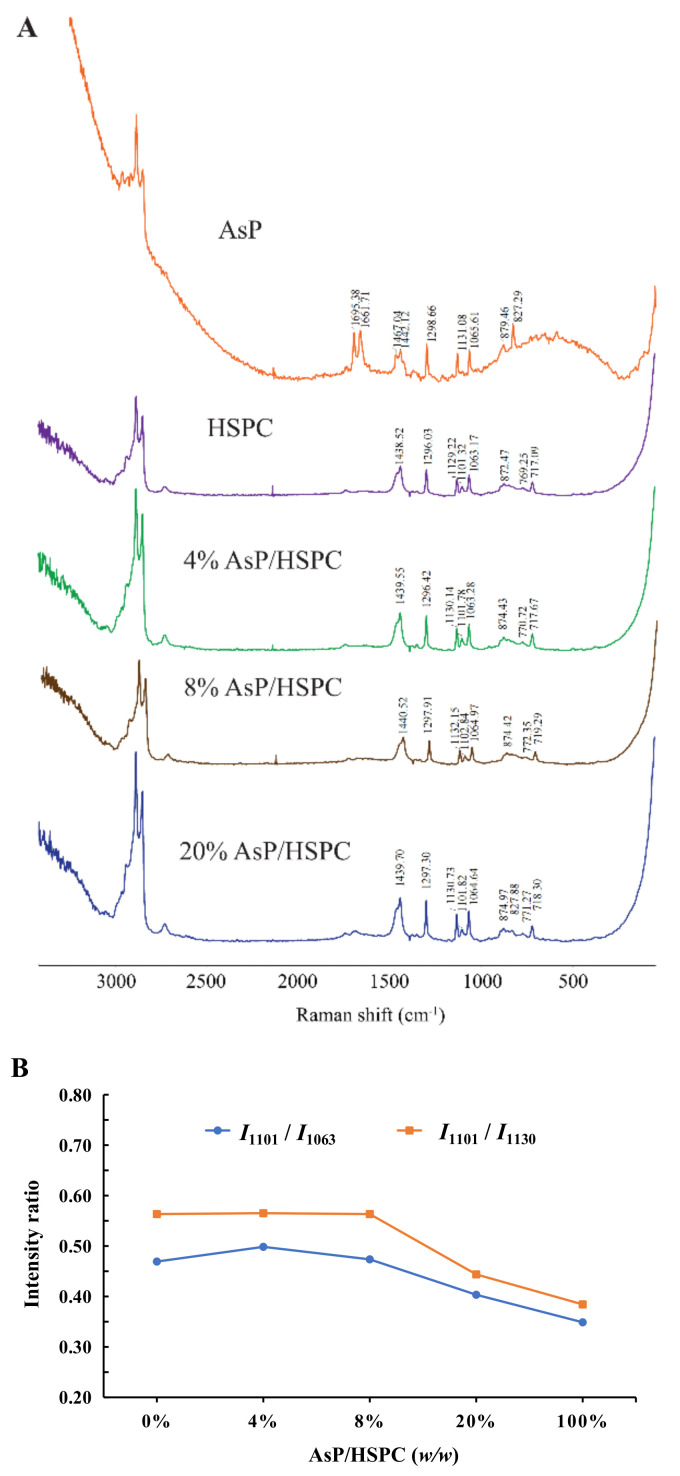
(**A**) Raman spectra of AsP–PC hydrates with various AsP/HSPC ratios (*w/w*); (**B**) the intensity ratios of *I*_1101_/*I*_1063_ and *I*_1101_/*I*_1130_ in Raman spectra.

Raman spectroscopy is a powerful tool for the investigation of lipid skeletal conformations [48]. This nondestructive technique has no water interference [49]. Figure 5A shows the Raman spectrums of AsP–PC hydrates with various AsP proportions. The characteristic broad Raman double-peak band of AsP lactone *v* (C=O) stretch at 1661–1695 cm^−1^ was not clearly identified because of the relatively low content of AsP in AsP–PC hydrates. Therefore, the effect of AsP on HSPC characteristic bands in the Raman spectrum are mainly discussed here. 

Three characteristic Raman bands for the fatty acid *v*(C–C) stretch at 1060–1140 cm^−1^ were analyzed to determine the effects of AsP on the conformational order of the lipid chains in AsP–PC hydrates. The bands at 1063 cm^−1^ and 1130 cm^−1^ were generated by the symmetric and asymmetric ordered *trans*-conformation *v*(C–C) stretches, respectively. The band at 1101 cm^−1^ was produced by the disordered gauche conformation *v*(C–C) stretch. The intensity ratios of the bands at 1101 cm^−1^ and 1063 cm^−1^ (*I*_1101_/*I*_1063_) and at 1101 cm^−1^ and 1130 cm^−1^ (*I*_1101_/*I*_1130_) indicate the disorder degree of the C–C skeleton of the fatty chain [50]. As shown in Figure 5B, *I*_1101_*/I*_1063_ and *I*_1101_/*I*_1130_ did not significantly differ from those for pure HSPC when the AsP/HSPC ratios were 4% (*w/w*) and 8% (*w/w*), respectively. However, *I*_1101_/*I*_1063_ and *I*_1101_/*I*_1130_ decreased when the AsP/HSPC ratio increased to 20% (*w/w*). Thus, the excessive AsP enhances the fatty chain ordering in AsP–PC hydrates. The band at 2800–3000 cm^−1^ was attributed to the fatty acid *v*(C–H) stretch and reflected its lattice stacking and structural changes [51,52]. However, the peaks in this region were noisy and misshapen. It was difficult to identify the structural changes in this case. The influence of AsP on the polar head group of HSPC was determined by the 717–772 cm^−1^ band range (Figure 4A). For the HSPC spectrum, the band at 717 cm^−1^ was ascribed to the *v*(C–N) stretch of the O–C–C–N^+^ skeleton in the gauche conformation, and the band at 770 cm^−1^ was caused by *v*(C–N) stretch of O–C–C–N^+^ in the *trans*-conformation [52]. The 717 cm^−1^ Raman shift was slightly red-shifted with gradual increase in AsP/HSPC ratio at no more than 8% (*w/w*). However, when the AsP/HSPC ratio continued to increase to 20% (*w/w*), the band at 717 cm^−1^ turned slightly blue-shifted. The 770 cm^−1^ peak intensity was weak for the AsP–PC hydrates, but the trend in the change of Raman shift was consistent with that for 717 cm^−1^. Hence, a low proportion AsP (≤8%, *w/w*) made the polar HSPC head groups slightly disordered and loose in the AsP–PC hydrates, while excessive AsP enhanced the order of the polar HSPC head. These results were consistent with those for DSC.

### 3.5. FTIR Spectroscopy

**Figure 6:**
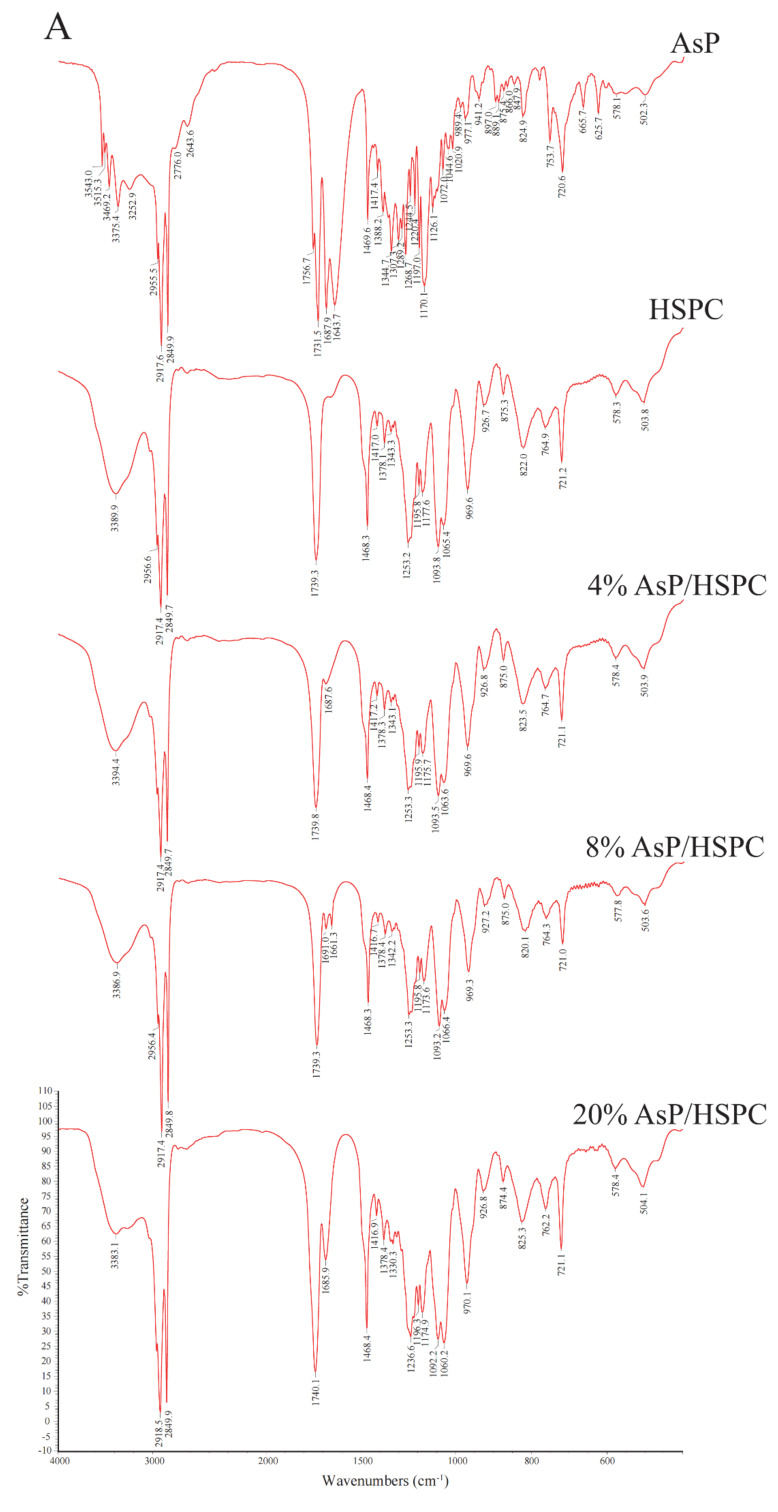

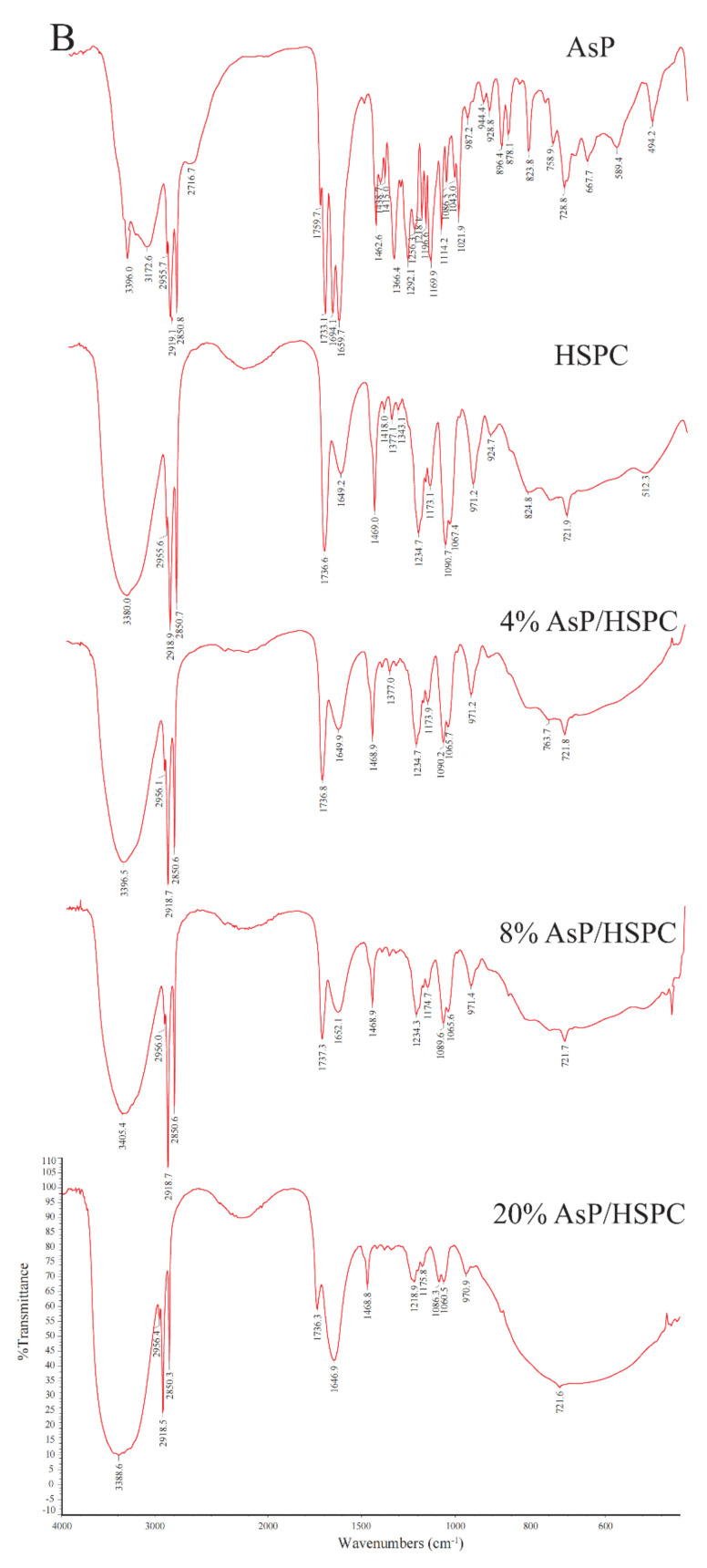
FTIR spectra of AsP and HSPC, and those of AsP–PC composites with various AsP/HSPC ratios (4%, 8%, 20%, *w*/*w*): (**A**) dry form; (**B**) hydrates.

FTIR provides submolecular-level conformational data about the lipids including the domains of the fatty acyl chains, polar headgroups, and glycerol backbones around the polar/nonpolar interface [53]. IR spectra for the dry and hydrated AsP–PC composites at various AsP/HSPC ratios are shown in Figure 6. AsP had only weak IR intensity because of its low content in the composite films. Therefore, we focused on the effects of AsP on the characteristic phospholipid bands in the IR spectrum.

#### 3.5.1. Effects of AsP on HSPC Acyl Chains

**Figure 7:**
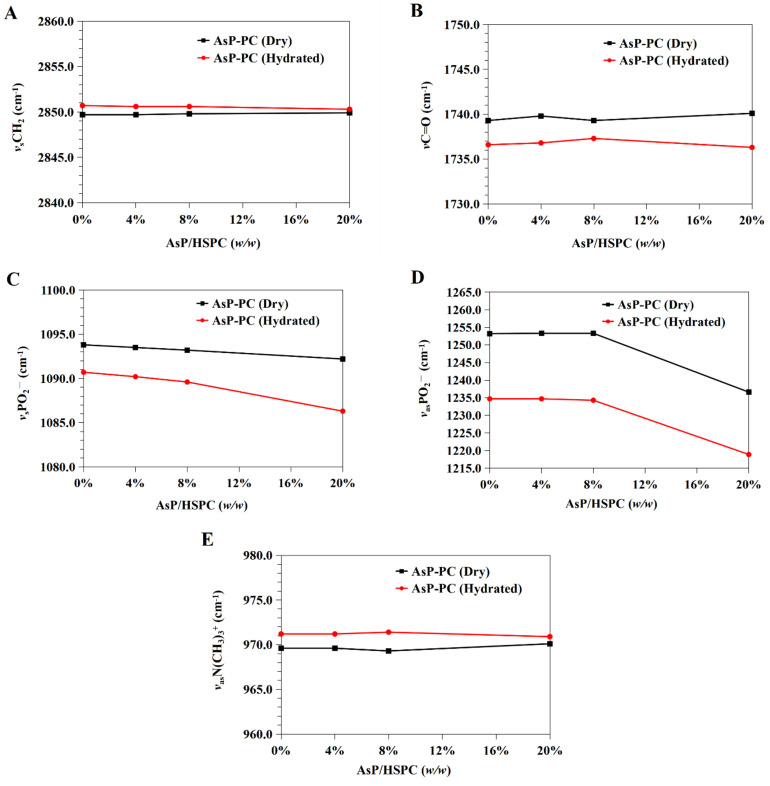
Infrared wavenumber displacement dependence on AsP proportion in the FTIR spectra of the dry and hydrated AsP–PC composites: (**A**) the CH_2_ symmetric stretching band (*ν*_s_CH_2_); (**B**) the C=O stretching band (*ν*C=O); (**C**) the symmetric stretching band of PO^2−^ (*ν*_s_PO^2−^); (**D**) the antisymmetric stretching band of PO^2−^ (*ν*_as_PO^2−^); (**E**) the antisymmetric stretching band of the choline group (*ν*_as_N(CH_3_)_3_^+^).

The CH_2_ symmetric stretching band *(ν*_s_CH_2_) at the phospholipid fatty acyl chains was ~2850.0 cm^−1^. A spectral blue shift in the maximum wavenumber of the CH_2_ symmetric stretching bands indicates an increase in chain disorder [54,55]. Figure 7A shows the wavenumber variation in this region for the dry and hydrated AsP–PC composites with various AsP proportions. The AsP amount did not markedly change in the *ν*_s_CH_2_ wavenumbers of AsP–PC composites, indicating that it had no obvious effect on the hydrophobic fatty acyl chains. All wavenumbers for *ν*_s_CH_2_ of AsP–PC hydrates were somewhat higher than those for the corresponding dry films; possibly the CH_2_ groups in the fatty acyl chains became slightly disordered in the presence of water.

#### 3.5.2. Effects of AsP on the Polar/Nonpolar PC Interface

The C=O groups in the glycerol backbones were at the polar/nonpolar interfaces and the hydrophobic/hydrophilic boundaries of the phospholipids [56]. Therefore, the C=O stretching band (*ν*C=O) at ~1735 cm^−1^ is important in investigations of the hydration degree of carbonyl groups, interactions with exogenous substances, and lipid conformational changes and phase transitions [53,56]. Figure 7B shows the *ν*C=O wavenumbers for the dry and hydrated AsP–PC composites with various AsP proportions. With gradual increase in AsP/HSPC ratio, the *ν*C=O wavenumbers for AsP–PC composites had a slight irregular fluctuation by <0.9 cm^−1^, showing no significant change. After hydration of AsP–PC composites with various ratios, all of the *ν*C=O wavenumbers were slightly decreased, possibly indicating there were measurable increases in carbonyl group hydration.

#### 3.5.3. Effects of AsP on the Polar PC Head

##### Effects of AsP on the PO^2−^ Group

The symmetric and antisymmetric stretching bands of PO^2−^ at the polar HSPC head were ~1090 cm^−1^ (*ν*_s_PO^2−^) and ~1230 cm^−1^ (*ν*_as_PO^2−^), respectively [57]. After hydration, the PO^2−^ wavenumber shifts reflected the hydration degree of this group, especially in the antisymmetric PO^2−^ stretching mode (*ν*_as_PO^2−^) [42,53]. As PO^2−^ is strongly electronegative, it could also form intermolecular hydrogen bonds with the polar groups of other molecules. Here, however, we focused primarily on the interactions with the ascorbate group of AsP. The wavenumbers for the *ν*_s_PO^2−^ and *ν*_as_PO^2−^ of AsP–PC composites with various AsP proportions are shown in Figure 7C and 7D, respectively, and are compared for the dry and hydrated states.

Figure 7C shows that the *ν*_s_PO^2−^ wavenumber of the dry AsP–PC composites gradually decreased with increasing AsP addition. Figure 7D shows that AsP addition did not markedly change *ν*_as_PO^2−^ when the AsP/HSPC proportion was ≤8% (*w/w*). However, when the AsP/HSPC proportion increased to 20% (*w/w*), the *ν*_as_PO^2−^ wavenumber sharply decreased by ~16 cm^−1^ below the maximum intensity. The apparent changes in the *ν*_s_PO^2−^ and *ν*_as_PO^2−^ for AsP–PC composites may be explained by direct hydrogen bonds between the polar head of AsP and the PO^2−^ group of HSPC, and the modes of hydrogen bonds changed differently when AsP addition was excessive. For AsP–PC hydrates, both *ν*_s_PO^2−^ and *ν*_as_PO^2−^ decreased in response to the hydration, and there was a strong shift of 17–20 cm^−1^ for *ν*_as_PO^2−^ (Figure 7D). The *ν*_s_PO^2−^ wavenumber notably declined more significantly after hydration when the AsP/HSPC proportion was increased to 20% (*w/w*), indicating that the effect of excessive Asp makes the PO^2−^ group more susceptible to hydration.

##### Effects of AsP on the N(CH_3_)_3_^+^ Group

The symmetric and antisymmetric stretching bands of the choline group [N(CH_3_)_3_^+^] in the polar heads of the phospholipids were ~920 cm^−1^ (*ν*_s_N(CH_3_)_3_^+^) and ~970 cm^−1^ (*ν*_as_N (CH_3_)_3_^+^), respectively [57]. Figure 6 shows that *ν*_s_N(CH_3_)_3_^+^ was not clearly identified in the hydrated AsP–PC spectrum because of strong interference from water. In this study, we focused on changes in *ν*_as_N(CH_3_)_3_^+^ as it is sensitive to dipolar interactions with water [52,54]. Figure 7E shows that the *ν*_as_N(CH_3_)_3_^+^ did not markedly change for the dry AsP–PC composites relative to HSPC with the AsP/HSPC ratio increase. The *ν*_as_N(CH_3_)_3_^+^ wavenumbers fluctuated slightly, less than 0.8 cm^−1^. For AsP–PC hydrates, all of the *ν*_as_N(CH_3_)_3_^+^ wavenumbers with various AsP proportions were somewhat higher than those for the corresponding dry films, indicating the hydration effect by water. However, different AsP proportions had little effect on the *ν*_as_N(CH_3_)_3_^+^ wavenumber change of AsP–PC hydrates.

### 3.6. Interactions between AsP and HSPC in AsP–PC Composites Modeled by Materials Studio

**Figure 8:**
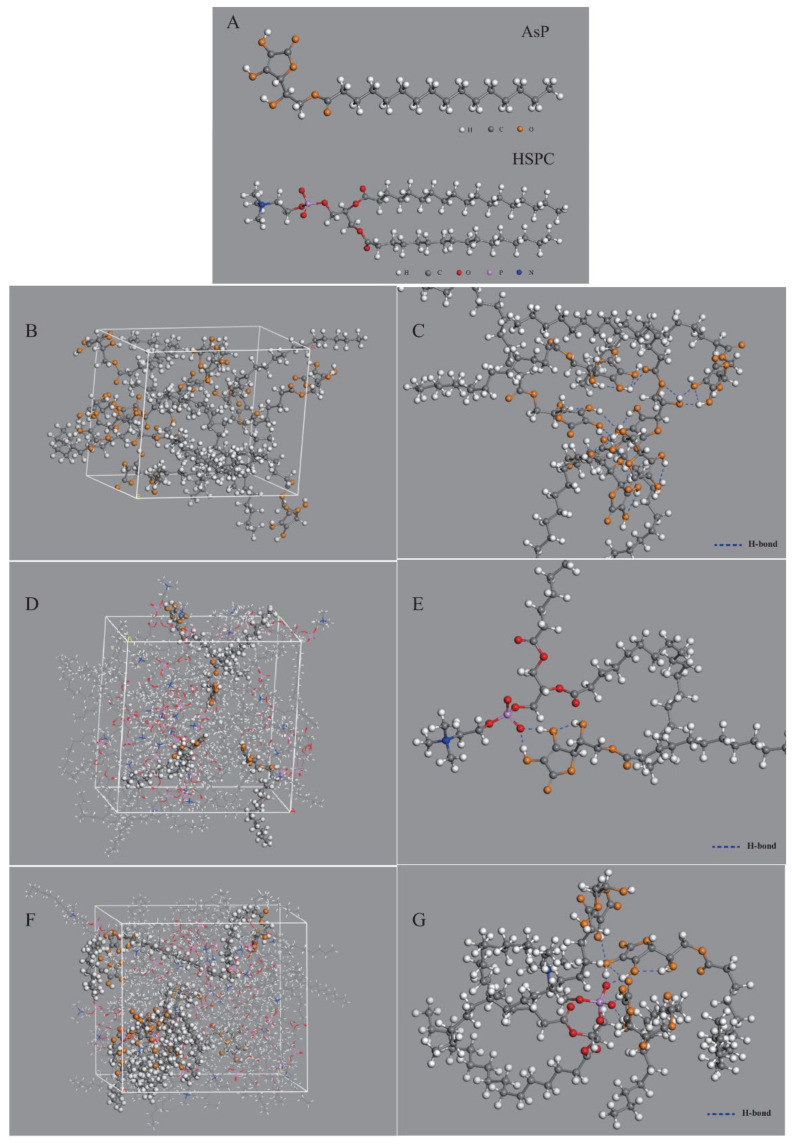
Computer models of molecular interaction between AsP and PC in AsP–PC composites by molecular dynamics simulation: (**A**) 3D structure of AsP and HSPC molecules; (**B**) the simulated single-component AsP model; (**C**) molecular interactions in the single component AsP model (the hydrogen bonds are given in blue dash lines); (**D**) the simulated AsP–PC model with 8 wt% AsP/HSPC (HSPC molecules are displayed in concise patterns by lines to distinguish it from AsP); (**E**) molecular interactions between AsP and HSPC in the AsP–PC model with 8 wt% AsP/HSPC; (**F**) the simulated AsP–PC model with 20 wt% AsP/HSPC (HSPC molecules are displayed in concise patterns by lines to distinguish it from AsP); (**G**) molecular interactions between AsP and HSPC in the AsP–PC model with 20 wt% AsP/HSPC.

Molecular dynamics was used to simulate 3D models of AsP–PC composites with various AsP proportions and elucidate the intermolecular interactions between AsP and HSPC (Figure 8A) without the influences of water and solvents.

Figure 8B shows the simulated single-component AsP model. The polar heads of AsP molecules were very close and their fatty acid chains were intertwined. Numerous hydrogen bonds were detected in or between the polar AsP heads (Figure 8C). Multiple hydroxyl groups in the ascorbic acid region formed hydrogen bonds in the polar heads especially among the enolic hydroxyl groups on the furan rings. The intermolecular hydrogen bonds were ~1.7–2.4 Å long, while the intramolecular hydrogen bonds were ~2.0–2.4 Å long.

Figure 8D shows the simulated AsP–PC model with AsP/HSPC ratio at 8% (*w/w*). The AsP molecules were uniformly distributed throughout the phospholipids. The polar heads of AsP and HSPC were close to each other, and their fatty chains were intertwined. Strong hydrogen bonds were formed between the enolic hydroxyl groups in AsP and the PO^2−^ of HSPC in the polar head region (Figure 8E). These intermolecular hydrogen bonds were ~1.4–1.6 Å long, shorter than those between AsP molecules. This could be explained by the fact that the enolic hydroxyl group in AsP readily interacts with the PO^2−^ of HSPC and forms stable intermolecular interactions because PO^2−^ has high electronegativity. Hence, AsP was well compatible and physically stable in combination with HSPC in AsP–PC composites when the AsP proportion was ≤8% (*w/w*). This simulation result was corroborated by the DSC results.

Figure 8F shows the simulated AsP–PC model with AsP/HSPC ratio at 20% (*w/w*). The AsP molecules were inhomogeneously distributed in HSPC, and there were AsP microaggregates in the simulated AsP–PC model. In the AsP microaggregate region (shown in Figure 8G), the types of hydrogen bonds between AsP and HSPC were considerably different from those in the low AsP proportion model (8%, *w/w*). The PO^2−^ of one HSPC molecule could simultaneously form hydrogen bonds with the enolic hydroxyl groups in multiple AsP molecules. Moreover, the adjacent AsP molecules could form hydrogen bonds with each other. The H-bond form change caused by excessive Asp strongly affected the physicochemical properties of AsP–PC composites. This was confirmed by the results of experiments, including DSC, Raman and FTIR spectra. Excess AsP was detrimental to the uniformity of the lipid film and was supposed to alter the physicochemical stability of liposomes and drug leakage. The present study empirically demonstrated that an AsP/HSPC ratio of ≤8% (*w/w*) was suitable for use in the liposome surface charge modification. 

### 3.7. Physicochemical Characterization of PTX-LNPs

Particle size is one of the most important factors in a particle drug delivery system. As shown in Table 2, the average particle sizes of the prepared PTX-LNP formulations were 86.9 ± 5.0, and 97.1 ± 0.3 nm for blank-LNPs. The PDIs of all formulations were less than 0.3, which indicates that the preparations had an ideal and narrow distribution and followed unimodal normal distribution (Figure 2).

The TEM image of PTX-LNPs is also shown in Figure 2, which demonstrates that PTX-LNPs are spherical in shape with an average size of about 100 nm.

The zeta potential indicates the degree of repulsion between similarly charged, adjacent particles in dispersion, and a higher zeta potential will confer stability and prevent aggregation. Table 2 shows the mean zeta potential of PTX-LNPs and blank-LNPs. The zeta potential values of PTX-LNPs and blank-LNPs were −44.3 ± 4.1 mV and −47.4 ± 0.6 mV, respectively, similar to those of the AsP-PC-SLNs.

### 3.8. Drug Loading (DL) and Encapsulation Efficiency (EE)

The DL and EE were determined by HPLC methods. Linear range, repeatability, and recovery were calculated under optimal conditions. To estimate the repeatability, shown as relative standard deviation percentage (% RSD), six replicates were employed, and the % RSD value was found to be less than 1.0%. Satisfactory recoveries (99.8%) and low RSD (less than 2.0%) were achieved, and good linearity was obtained (*r*^2^ > 0.9999). Table 2 shows the EE and DL of PTX-LNPs. The results indicate that the membrane composed of AsP–PC composites could well encapsulate PTX, the EE was close to 100%, and the DL was almost equal to the theoretical value.

### 3.9. In Vitro Cytotoxicity Analysis

**Figure 9:**
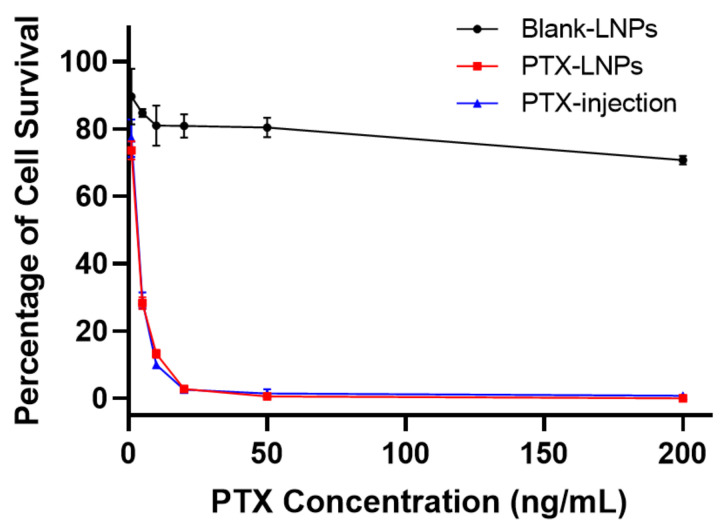
In vitro cytotoxicity evaluation of PTX-LNPs, PTX injection, and blank-LNPs in MDA-MB-453 cells.

To compare the cytotoxic differences among the PTX-LNPs, PTX-injection, and blank-LNPs against the MDA-MB-453 cell line, the in vitro cytotoxicity activity was investigated using the CCK8 assay. Figure 9 shows the cell viability following 72 h of incubation at series PTX concentrations. The results show that the blank-LNPs failed to exhibit cytotoxicity in the MDA-MB-453 cells, and the viability was not less than 80%, indicating the composition used to formulate LNPs was safe and biocompatible in vivo. In addition, PTX-LNPs exhibited antitumor activity in vitro similar to that of PTX injection.

### 3.10. In Vivo Anti Breast Cancer Effects of PTX-LNPs

**Figure 10:**
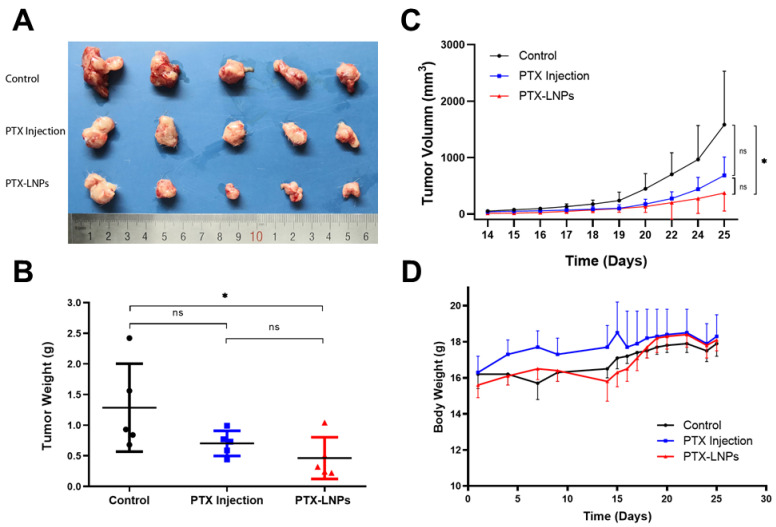
Anti-breast cancer efficacy of PTX-LNPs in 4T1 orthotropic mice. Notes: (**A**) images for the tumor of control, PTX injection and PTX-LNPs groups; (**B**) tumor weight statistics for the control, PTX injection, and PTX-LNPs groups; (**C**) variation of tumor volume for the control, PTX injection, and PTX-LNPs groups; (**D**) body weights for the control, PTX injection, and PTX-LNPs groups. (*n* = 5) Abbreviations: PTX, Paclitaxel; PTX-LNPs, Paclitaxel lipid nanoparticles. Significant differences are indicated: * *p* < 0.05, ns (no significance). black bullet (Control), blue square (PTX Injection), red triangle (PTX-LNPs).

The in vivo anti breast cancer efficacy was evaluated in female BALB/c mice bearing 4T1 orthotropic breast cancer. The tumor weight, tumor volume, and, body weight were monitored and quantified at predetermined time points to evaluate the tumor inhibitory effects of PTX-LNPs, as shown in Figure 10A–C. The results showed that PTX injection had a relatively obvious antitumor effect, but there was no statistical difference compared with the control group. Compared with the control group, PTX-LNPs had obvious anti-tumor effects, and the results were statistically different. The therapeutic effect of PTX-LNPs was better than that of PTX injection, but the results did not show a statistical difference. The reason may be due to an insufficient sample amount (*n* = 5). There were no significant differences in the body weights of the three groups of animals administered PBS, PTX injection, and PTX-LNPs (Figure 10D).

**Figure 11:**
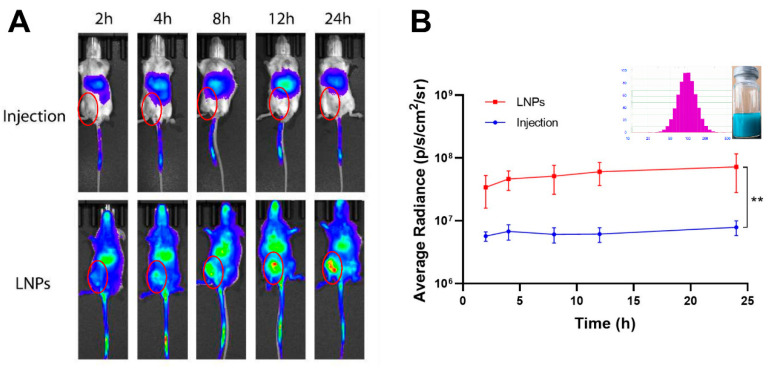
Breast cancer targeted efficacy of LNPs in 4T1 orthotropic mice. Notes: (**A**) in vivo fluorescence signal changes of the 4T1 tumor-bearing mice as the time passed after intravenous injection of normal DiR injection or DiR-LNPs; (**B**) quantitative image analysis of the intensity of light emitted from tumor sites (*n* = 3). Abbreviations: DiR, 1,1′-dioctadecyl-3,3,3′,3′-tetramethylindotricarbocyanine iodide; PTX, Paclitaxel; LNPs, lipid nanoparticles. Significant differences are indicated: ** *p* < 0.01.

In order to observe the tumor targeted efficacy of the LNPs, the DiR fluorescence signals were monitored and quantified at predetermined time points, as shown in Figure 11. The results showed that the drugs were mainly distributed to the lungs after intravenous normal injection. Compared with the normal injection, most of the LNPs were trapped in the tumor tissue after intravenous injection. Moreover, along with the extension of treatment time, the LNPs could significantly increase drug accumulation in tumor tissues. This selective tumor tissue target ability may be relied on for EPR effect, whose mechanism was based on the pathophysiological features of tumor tissue.

## 4. Conclusions

In the present study, AsP served as a negatively charged lipid to modify the surface charge of lipid nanocarriers. We used various analytical methods and MD computer simulation to investigate the physicochemical properties of the AsP-PC-LNPs and the interactions between AsP and PC. The results revealed that the ionized AsP was the substance basis for modifying the surface charge, and the zeta potential was correlated with the amount and distribution of AsP on the surface at neutral pH. AsP was well compatible and physically stable in combination with PC in AsP–PC composites when the AsP/PC ratio was ≤8% (*w/w*). However, when the AsP/PC ratio was >8% (*w/w*), the excessive AsP changed the H-bond form between AsP and PC, and was detrimental to the uniformity of the film of nanoparticles, which strongly affected the physicochemical properties of AsP-PC-LNPs and was supposed to alter the physicochemical stability of nanoparticles. This work helped to clarify the mechanism of surface charge modification by AsP and provided a theoretical foundation for the rational use of AsP as a charged lipid to improve surface properties of nanoparticles, which is important in improving the efficiency of targeted drug delivery. Furthermore, AsP–PC composites were used as PBBM to prepare PTX-LNPs, which have stable surface negative charge, better tumor targeting and tumor inhibitory effects.

## Figures and Tables

**Table 1:** Effect of AsP/HSPC ratios on zeta potential and particle size of AsP-PC-LNPs (*n* = 3).

AsP/HSPC (%, *w/w*)	Zeta (mV) (Average ± S.D.)	Particle Size (nm) (Average ± S.D.)	PDI (Average ± S.D.)
0%	−2.6 ± 0.0	87.6 ± 0.7	0.411 ± 0.017
1%	−29.2 ± 0.3	105.2 ± 0.5	0.300 ± 0.005
4%	−33.9 ± 1.5	73.7 ± 0.1	0.281 ± 0.009
8%	−42.5 ± 0.2	98.7 ± 1.0	0.259 ± 0.016
12%	−43.3 ± 0.1	99.3 ± 1.1	0.208 ± 0.014

**Table 2:** The Physicochemical Characterization of PTX-LNPs and LNPs.

Sample	Particle Size (nm)	PDI	Zeta (mV)	EE (%)	DL (%)
PTX-LNPs	86.9 ± 5.0	0.166 ± 0.014	−44.3 ± 4.1	98.7 ± 0.6	0.987 ± 0.006
Blank-LNPs	97.1 ± 0.3	0.173 ± 0.010	−47.4 ± 0.6	/	/

## Data Availability

All data are fully available without restriction.

## Sample Availability

Samples of the compounds are available from the authors.

## Abbreviations

API	active pharmaceutical ingredient
AsP	ascorbyl palmitate
AsP-PC	composite lipids prepared by AsP and HSPC
AsP-PC-LNPs	lipid nanoparticles prepared by AsP and HSPC
AsP-free-LNPs	lipid nanoparticles prepared by HSPC alone
CVFF	consistent-valence force field
DiR	1,1′-dioctadecyl-3,3,3′,3′-tetramethylindotricarbocyanine iodide
DL	drug loading
DLS	dynamic light scattering
DSC	differential scanning calorimetry
EE	encapsulation efficiency
FTIR	Fourier transform infrared spectroscopy
H-bond	hydrogen bond
HSPC	hydrogenated soy phosphatidylcholine
LNPs	lipid nanoparticles
MD	molecular dynamics
MPS	mononuclear phagocyte system
NPT	isothermal-isobaric ensemble
NVT	canonical ensemble
PBBM	phospholipids based biological membranes
PC	phosphatidylcholine
PTX	paclitaxel
PTX-LNPs	paclitaxel loaded lipid nanoparticles
